# Association between exercise frequency with renal and cardiovascular outcomes in diabetic and non-diabetic individuals at high cardiovascular risk

**DOI:** 10.1186/s12933-021-01429-w

**Published:** 2022-01-20

**Authors:** Michael Böhm, Helmut Schumacher, Christian Werner, Koon K. Teo, Eva M. Lonn, Felix Mahfoud, Thimoteus Speer, Giuseppe Mancia, Josep Redon, Roland E. Schmieder, Karen Sliwa, Nikolaus Marx, Michael A. Weber, Ulrich Laufs, Bryan Williams, Salim Yusuf, Johannes F. E. Mann

**Affiliations:** 1 Klinik für Innere Medizin III, Kardiologie, Angiologie und Internistische Intensivmedizin, Universitätsklinikum des Saarlandes, Saarland University, Kirrberger Str. 1, 66421 Homburg/Saar, Germany; 2 Statistical Consultant, 55218 Ingelheim, Germany; 3 Department of Medicine, Population Health Research Institute, McMaster University, Hamilton, ON L8L 2X2 Canada; 4 Klinik für Innere Medizin IV, Universitätsklinikum des Saarlandes, Saarland University, Kirrberger Str.1, Homburg/Saar, Germany; 5 University of Milano-Bicocca, Istituto Clinico Universitario Policlinico di Monza, Piazza dell’Ateneo Nuovo, 1, Milan, Italy; 6 Hypertension Unit, Hospital CIínico Universitario, University of Valencia, Av. de Blasco Ibáñez, 13, València, Spain; 7 CIBERObn, Institute of Health Carlos III, Madrid, Spain; 8 Department of Nephrology and Hypertension, University Hospital, Friedrich-Alexander University, Erlangen/Nuremberg, Germany; 9 Faculty of Health Sciences, Hatter Institute for Cardiovascular Research in Africa & IIDMM, University of Cape Town, Cape Town, South Africa; 10 Department of Internal Medicine, University Hospital RWTH Aachen, Pauwelsstraße 30, Aachen, Germany; 11 Downstate College of Medicine, State University of New York, 450 Clarkson Ave, Brooklyn, NY USA; 12 Klinik und Poliklinik für Kardiologie, Universitätsklinikum Leipzig, Liebigstr. 20, 04103 Leipzig, Germany; 13 University College London (UCL), Institute of Cardiovascular Science, National Institute for Health Research (NIHR), UCL Hospitals Biomedical Research Centre, 149 Tottenham Court Road, London, UK; 14 KfH Kidney Centre, München-Schwabing, Minich, Germany; 15 Department of Nephrology and Hypertension, University Hospital, Friedrich-Alexander University, Schlossplatz 4, Erlangen, Germany

**Keywords:** Physical activity, Cardiovascular outcomes, Renal outcomes, Secondary prevention

## Abstract

**Background:**

Guidelines recommend physical activity to reduce cardiovascular (CV) events. The association between physical activity and progression of chronic kidney disease (CKD) with and without diabetes is unknown. We assessed the association of self-reported physical activity with renal and CV outcomes in high-risk patients aged ≥ 55 years over a median follow-up of 56 months in post-hoc analysis of a previously randomized trial program.

**Methods:**

Analyses were done with Cox regression analysis, mixed models for repeated measures, ANOVA and χ^2^-test. 31,312 patients, among them 19,664 with and 11,648 without diabetes were analyzed.

**Results:**

Physical activity was inversely associated with renal outcomes (doubling of creatinine, end-stage kidney disease (ESRD)) and CV outcomes (CV death, myocardial infarction, stroke, heart failure hospitalization). Moderate activity (at least 2 times/week to every day) was associated with lower risk of renal outcomes and lower incidence of new albuminuria (p < 0.0001 for both) compared to lower exercise levels. Similar results were observed for those with and without diabetes without interaction for renal outcomes (p = 0.097–0.27). Physical activity was associated with reduced eGFR decline with a moderate association between activity and diabetes status (p = 0.05).

**Conclusions:**

Moderate physical activity was associated with improved kidney outcomes with a threshold at two sessions per week. The association of physical activity with renal outcomes did not meaningfully differ with or without diabetes but absolute benefit of activity was even greater in people with diabetes. Thus, risks were similar between those with diabetes undertaking high physical activity and those without diabetes but low physical activity.

*Clinical trial registration*: http://clinicaltrials.gov.uniqueidentifier:NCT00153101.

**Supplementary Information:**

The online version contains supplementary material available at 10.1186/s12933-021-01429-w.

## Background

Chronic kidney disease (CKD) is among the top 10 non-communicable conditions associated with high morbidity and mortality [1] affecting ~ 10% of the world population [1, 2]. Progression of CKD is accelerated by comorbidities and unhealthy lifestyle such as diabetes, high blood pressure, unhealthy diet and physical inactivity [3–5]. These associations tend to be stronger in subjects with diabetes [6] who accumulate a high number of cardiovascular (CV) and renal events [7]. Physical fitness is associated with lower risk for atherosclerotic CV events compared to a sedentary lifestyle in the general population [8, 9]. Current guidelines recommend active lifestyles to reduce the risk of CV events [10–12], mainly based on systematic meta-analyses of small prospective cohort studies [13]. Previous studies suggested that exercise is associated with improved CV outcomes [14]. However, less literature deals with the time course of CKD progression and exercise [3] and differences between patients with and without diabetes. Renal outcomes were key secondary endpoints in The Ongoing Telmisartan Alone and in Combination With Ramipril Global Endpoint Trial (ONTARGET) [15] and the Telmisartan Randomised AssessmeNt Study in ACE iNtolerant subjects with cardiovascular Disease (TRANSCEND) [16] trials which randomized high risk CV patients to ramipril, telmisartan or both of these drugs with neutral CV results in all treatment strata [15–17]. As this trial program recorded self-reported exercise at enrollment and adjudicated rigorously endpoints, it represents a unique database to investigate the associations of physical activity with renal outcomes in 31,312 patients with approximately a third with a history of diabetes. Patients were randomized to ACEi or ARB or both in ONTARET and to ARB or placebo in TRANSCEND. Randomized treatments had no significant effect on renal of CV outcomes [15–17]. Hence, the treatment groups were pooled and analyzed together in this post-hoc analysis.

## Methods

### Study design and population

**Fig. 1:**
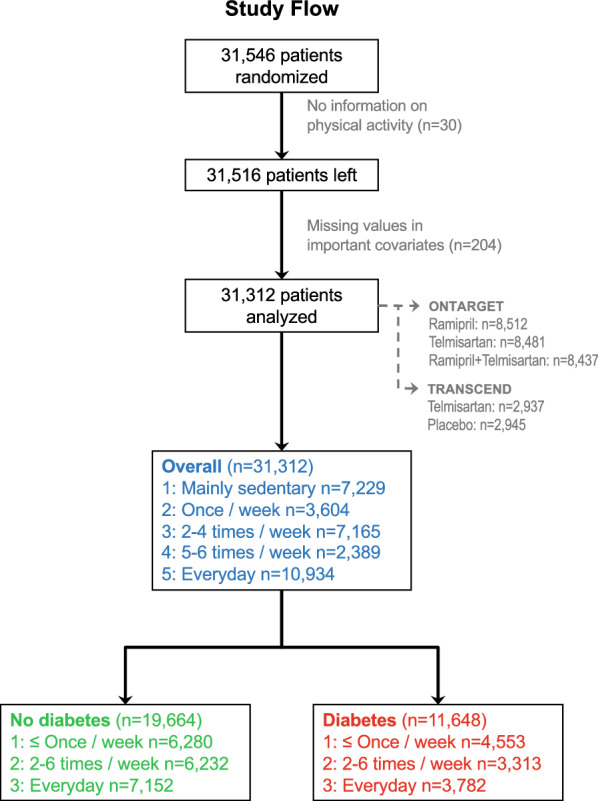
Study flow of patients included in the analysis and treatment allocation in the ONTARGET/TRANSCEND trials

In ONTARGET/TRANSCEND, patients without symptomatic heart failure were recruited from 737 centers in 40 countries with a median of follow-up of 56 months. The population consisted of patients with high CV risk defined as a history of coronary artery disease with a previous myocardial infarction or peripheral artery disease or transient ischemic attack or stroke or diabetes mellitus complicated by end-organ damage. If diabetes was the inclusion criterion (i.e. no previous CV event), evidence of end-organ damage was defined as retinopathy, left ventricular hypertrophy, or macro- or microalbuminuria. The design, treatments, algorithms and the results of ONTARGET and TRANSCEND have been reported previously [15–17]. Patients were randomly assigned to ramipril, telmisartan or a combination of ramipril and telmisartan for the duration for the study. Continuation of anti-hypertensive medications and adjustment of blood pressure treatments if not controlled was mandated. As there were no differences of CV [15, 16] and renal [17] outcomes between the randomized treatment groups, patients were pooled allowing an adequately powered, comprehensive post-hoc analysis of the association of renal and CV outcomes (for comparison) according to self-reported physical activity levels. Only patients with complete data entered the analysis. The study flow, censoring criteria, and trial or treatment allocations of the present report are summarized in Fig. 1. Of 31,546 patients randomized, 30 patients were censored for missing data on physical activity and 204 for missing values of important covariables. 31,312 patients entered the present analysis, 19,664 patients without diabetes and 11,648 patients with diabetes. Clinical diagnostic criteria for diabetes were fasting glucose ≥ 7 mmol/l, elevated HbA1C to ≥ 110% of upper limit norm of the study center, the initiation of insulin or oral hypoglycaemic patients and/or a 2-h glucose ≥ 11.1 mmol/l following a 75 g oral glucose tolerance test. For patients with diabetes only recruited into the studies, evidence of end organ damage as retinopathy, left ventricular hypertrophy, macro- or microalbuminuria or any evidence of previous cardiac or vascular disease had to be present.

### Description of physical activity

In the clinical research file (CRF), participants indicated their usual level of physical activity according to five activity levels at the randomization visit (mainly sedentary, once/week, 2–4 times/week, 5–6 times/week and daily). The choices were:

“How often do you engage in physical activity? (choose one)

o mainly sedentary
o  < once/week
o 2-4 times/week
o 5-6 times/week
o everyday”

We decided, before starting this analysis, to group all participants according to three levels of activity (≤ once/week, 3–6 times/week and every day) to enhance statistical power. Subgroups with or without diabetes, and with or without a history of stroke, myocardial infarction, or both or with different Framingham risk scores were also analyzed according to exercise levels.

### Outcomes

The primary CV outcome was a composite of CV death, myocardial infarction, stroke, or hospitalization for heart failure as published previously [15, 16]. All primary and secondary outcome events were adjudicated by a blinded central committee according to standard criteria [15, 16]. For renal outcomes, only patients with baseline information on serum creatinine level were included. Baseline serum creatinine level and baseline urinary albumin-creatinine ratio before the run-in phase as baseline were measured at a central laboratory with standard methods [17]. Microalbuminuria was defined as 30 mg/g creatinine to less than 300 mg/g creatinine. Macro-albuminuria was defined as 300 mg/g creatinine or greater. Estimated glomerular filtration rate (eGFR) was determined according to the Chronic Kidney Disease Epidemiology Collaboration (CKD-EPI) equation. We analyzed change of eGFR from baseline to week 260 and the chronic slope of the change in eGFR on treatment from week 6 to week 260. Renal outcomes were defined as end-stage renal disease (ESRD) or doubling of serum creatinine from baseline. The protocols were approved by the local ethic committees of each participating center and the regulatory authorities in each country. Ethics approval was obtained at each study site. Each participant gave written informed consent to the studies and their procedures.

### Statistical analysis

Groups were tested for differences using analysis of variance (ANOVA) for continuous data and chi square test for categorical data. Incident event curves were analyzed by physical activity levels and tested for differences using Cox regression, adjusting for baseline characteristics and important clinical confounders such as baseline systolic and diastolic blood pressure (SBP, DBP), heart rate (HR), age, sex, body mass index, baseline eGFR, geographical region, physical activity, formal education, alcohol consumption, tobacco use, history of hypertension, myocardial infarction, stroke, transient ischemic attack, heart rhythm, co-medications and study medications taking low physical activity levels (“mainly sedentary”) as reference (HR = 1). The changes of eGFR over time were analyzed in a mixed model for repeated measures (MMRM). Cox regressions were adjusted for competing risk of death. All analyses were done with SAS 9.4 (SAS Institute, NC, USA).

## Results

Recruitment for ONTARGET took place between December 1, 2001 and July 31, 2003; and for TRANSCEND between November 1, 2001 and May 30, 2004; 31,546 patients were randomized from 737 centers in 40 countries and followed-up for a median of 56 months.

Table 1 shows the demographic and clinical characteristics of the whole study population according to physical activity levels, categorized by mainly sedentary, once/week, 3–4 times/week, 5–6 times/week and every day physical activities (five categories). People with higher levels of exercise were less frequently smokers, had lower resting heart rate, higher baseline eGFR, less albuminuria and were younger compared to mainly sedentary individuals, while SBP was not different. Table 1 also displays subpopulations with or without diabetes. They were categorized into three groups of physical activity (≤ once/week, 3–6/week and every day). Blood pressure, heart rate, urine albumin excretion and body weight were higher in patients with than without diabetes.

**Table 1:** Baseline characteristics stratified by physical activity groups and diabetes status

		Physical activity					Total	p-value
		Mainly sedentary	Once/week	2–4times/week	5–6times/week	Everyday		
Number of patients	N	7229	3604	7156	23,89	10,934	31,312	
Baseline SBP	Mean (SD)	141.8 (17.2)	141.9 (17.2)	141.7 (17.3)	141.0 (17.2)	141.5 (17.3)	141.6 (17.3)	0.23
Baseline DBP	Mean (SD)	82.0 (10.5)	82.4 (10.1)	82.3 (10.3)	82.0 (10.3)	81.8 (10.3)	82.0 (10.3)	0.0059
Baseline RHR	Mean (SD)	70.2 (11.9)	69.2 (12.3)	67.3 (12.0)	66.1 (12.5)	67.2 (12.1)	68.1 (12.1)	<0.0001
Baseline eGFR (MDRD)	Mean (SD)	71.4 (21.9)	73.5 (20.5)	74.2 (19.1)	74.6 (18.6)	74.1 (19.4)	73.4 (20.0)	<0.0001
Baseline eGFR (CKD-EPI)	Mean (SD)	68.3 (18.6)	70.8 (17.9)	71.6 (16.6)	72.0 (16.5)	71.3 (16.7)	70.7 (17.3)	<0.0001
Age	Mean (SD)	67.3 (7.7)	66.2 (7.4)	66.0 (6.9)	66.2 (6.9)	66.5 (7.0)	66.5 (7.2)	<0.0001
Age group								<0.0001
<65 years	N (%)	2823 (39.1%)	1615 (44.8%)	3215 (44.9%)	1023 (42.8%)	4599 (42.1%)	13,275 (42.4%)	
≥ 65–< 75 years	N (%)	3023 (41.8%)	1454 (40.3%)	3029 (42.3%)	1058 (44.3%)	4768 (43.6%)	13,332 (42.6%)	
≥ 75 years	N (%)	1383 (19.1%)	535 (14.8%)	912 (12.7%)	308 (12.9%)	1567 (14.3%)	4705 (15.0%)	
Sex								<0.0001
Male	N (%)	4320 (59.8%)	2496 (69.3%)	5176 (72.3%)	1807 (75.6%)	8205 (75.0%)	22,004 (70.3%)	
Female	N (%)	2909 (40.2%)	1108 (30.7%)	1980 (27.7%)	582 (24.4%)	2729 (25.0%)	9308 (29.7%)	
Body mass index [kg/m^2^]	Mean (SD)	28.9 (5.5)	28.5 (5.0)	28.4 (4.6)	28.0 (4.3)	27.5 (4.3)	28.2 (4.8)	<0.0001
Obese	N (%)	2749 (38.0%)	1260 (35.0%)	2405 (33.6%)	693 (29.0%)	3162 (28.9%)	10,269 (32.8%)	<0.0001
Alcohol consumption	N (%)	2260 (31.3%)	1270 (35.2%)	3164 (44.2%)	1084 (45.4%)	4324 (39.5%)	12,102 (38.6%)	<0.0001
Tobaccouse, decode								<0.0001
Current	N (%)	1038 (14.4%)	533 (14.8%)	836 (11.7%)	212 (8.9%)	1162 (10.6%)	3781 (12.1%)	
Formerly	N (%)	3066 (42.4%)	1693 (47.0%)	3794 (53.0%)	1329 (55.6%)	5858 (53.6%)	15,740 (50.3%)	
Never	N (%)	3125 (43.2%)	1378 (38.2%)	2526 (35.3%)	848 (35.5%)	3914 (35.8%)	11,791 (37.7%)	
History of hypertension	N (%)	5507 (76.2%)	2566 (71.2%)	4926 (68.8%)	1583 (66.3%)	7401 (67.7%)	21,983 (70.2%)	<0.0001
Diabetes	N (%)	3124 (43.2%)	1429 (39.7%)	2539 (35.5%)	774 (32.4%)	3782 (34.6%)	11,648 (37.2%)	<0.0001
Myocardial infarction	N (%)	3173 (43.9%)	1756 (48.7%)	3575 (50.0%)	1273 (53.3%)	5419 (49.6%)	15,196 (48.5%)	<0.0001
Stroke/TIA	N (%)	1885 (26.1%)	667 (18.5%)	1277 (17.8%)	394 (16.5%)	2359 (21.6%)	6582 (21.0%)	<0.0001
Medication								
Aspirin	N (%)	5094 (70.5%)	2659 (73.8%)	5521 (77.2%)	1903 (79.7%)	8494 (77.7%)	23,671 (75.6%)	<0.0001
Beta-blockers	N (%)	3770 (52.2%)	2111 (58.6%)	4292 (60.0%)	1482 (62.0%)	6262 (57.3%)	17,917 (57.2%)	<0.0001
Diuretics	N (%)	2617 (36.2%)	1112 (30.9%)	1956 (27.3%)	612 (25.6%)	2751 (25.2%)	9048 (28.9%)	<0.0001
Nitrates	N (%)	2234 (30.9%)	1144 (31.7%)	1944 (27.2%)	708 (29.6%)	3455 (31.6%)	9485 (30.3%)	<0.0001
Other Ca^2+^ channel blockers	N (%)	1952 (27.0%)	866 (24.0%)	1694 (23.7%)	538 (22.5%)	2819 (25.8%)	7869 (25.1%)	<0.0001
Oral hypoglycemic agents	N (%)	2150 (29.7%)	928 (25.7%)	1665 (23.3%)	525 (22.0%)	2523 (23.1%)	7791 (24.9%)	<0.0001
Insulin	N (%)	879 (12.2%)	416 (11.5%)	674 (9.4%)	158 (6.6%)	921 (8.4%)	3048 (9.7%)	<0.0001
Statins	N (%)	3794 (52.5%)	2083 (57.8%)	4671 (65.3%)	1582 (66.2%)	6781 (62.0%)	18,911 (60.4%)	<0.0001
Number of antihypertensives								<0.0001
0	N (%)	1669 (23.1%)	801 (22.2%)	1652 (23.1%)	526 (22.0%)	2591 (23.7%)	7239 (23.1%)	
1	N (%)	3249 (44.9%)	1699 (47.1%)	3442 (48.1%)	1196 (50.1%)	5328 (48.7%)	14,914 (47.6%)	
2	N (%)	1843 (25.5%)	922 (25.6%)	1686 (23.6%)	565 (23.7%)	2541 (23.2%)	7557 (24.1%)	
3	N (%)	468 (6.5%)	182 (5.0%)	376 (5.3%)	102 (4.3%)	474 (4.3%)	1602 (5.1%)	

No diabetes—physical activity					Diabetes—physical activity					p-value No diabetes vs diabetes
≤Once/week	2-6times/week	Everyday	Total	p-value	≤Once/week	2-6times/week	Everyday	Total	p-value	
6280	6232	7152	19664		4553	3313	3782	11648		
140.8 (17.2)	140.3 (17.4)	140.6 (17.6)	140.6 (17.4)	0.20	143.2 (17.0)	143.8 (16.9)	143.4 (16.6)	143.4 (16.9)	0.27	<0.0001
82.4 (10.3)	82.2 (10.4)	82.0 (10.4)	82.2 (10.4)	0.17	81.8 (10.4)	82.3 (10.2)	81.4 (10.1)	81.8 (10.3)	0.0005	0.0017
68.2 (11.8)	65.5 (12.0)	65.6 (11.7)	66.4 (11.9)	<0.0001	72.1 (12.1)	69.8 (12.0)	70.2 (12.1)	70.8 (12.1)	<0.0001	<0.0001
72.7 (20.6)	74.6 (18.0)	74.0 (18.2)	73.7 (18.9)	<0.0001	71.3 (22.6)	73.8 (20.7)	74.2 (21.5)	72.9 (21.7)	<0.0001	0.0005
69.7 (17.6)	72.1 (15.9)	71.3 (16.0)	71.0 (16.5)	<0.0001	68.5 (19.5)	71.1 (17.8)	71.2 (18.1)	70.1 (18.6)	<0.0001	<0.0001
67.3 (7.9)	66.1 (7.1)	66.7 (7.2)	66.7 (7.4)	<0.0001	66.4 (7.2)	65.9 (6.6)	66.1 (6.8)	66.2 (6.9)	0.0020	<0.0001
				<0.0001					<0.0001	<0.0001
2515 (40.0%)	2758 (44.3%)	2954 (41.3%)	8227 (41.8%)		1923 (42.2%)	1480 (44.7%)	1645 (43.5%)	5048 (43.3%)		
2524 (40.2%)	2625 (42.1%)	3097 (43.3%)	8246 (41.9%)		1953 (42.9%)	1462 (44.1%)	1671 (44.2%)	5086 (43.7%)		
1241 (19.8%)	849 (13.6%)	1101 (15.4%)	3191 (16.2%)		677 (14.9%)	371 (11.2%)	466 (12.3%)	1514 (13.0%)		
				<0.0001					<0.0001	<0.0001
4188 (66.7%)	4779 (76.7%)	5580 (78.0%)	14,547 (74.0%)		2628 (57.7%)	2204 (66.5%)	2625 (69.4%)	7457 (64.0%)		
2092 (33.3%)	1453 (23.3%)	1572 (22.0%)	5117 (26.0%)		1925 (42.3%)	1109 (33.5%)	1157 (30.6%)	4191 (36.0%)		
27.9 (4.8)	27.6 (4.1)	27.0 (3.9)	27.5 (4.3)	<0.0001	30.0 (5.7)	29.4 (4.9)	28.4 (4.9)	29.3 (5.3)	<0.0001	<0.0001
1877 (29.9%)	1677 (26.9%)	1704 (23.8%)	5258 (26.7%)	<0.0001	2132 (46.8%)	1421 (42.9%)	1458 (38.6%)	5011 (43.0%)	<0.0001	<0.0001
2333 (37.1%)	3091 (49.6%)	3198 (44.7%)	8622 (43.8%)	<0.0001	1197 (26.3%)	1157 (34.9%)	1126 (29.8%)	3480 (29.9%)	<0.0001	<0.0001
				<0.0001					<0.0001	<0.0001
1022 (16.3%)	719 (11.5%)	800 (11.2%)	2541 (12.9%)		549 (12.1%)	329 (9.9%)	362 (9.6%)	1240 (10.6%)		
2913 (46.4%)	3474 (55.7%)	3968 (55.5%)	10,355 (52.7%)		1846 (40.5%)	1649 (49.8%)	1890 (50.0%)	5385 (46.2%)		
2345 (37.3%)	2039 (32.7%)	2384 (33.3%)	6768 (34.4%)		2158 (47.4%)	1335 (40.3%)	1530 (40.5%)	5023 (43.1%)		
4337 (69.1%)	3872 (62.1%)	4457 (62.3%)	12,666 (64.4%)	<0.0001	3736 (82.1%)	2637 (79.6%)	2944 (77.8%)	9317 (80.0%)	<0.0001	<0.0001
NA	NA	NA	NA	NA	NA	NA	NA	NA	NA	NA
3303 (52.6%)	3538 (56.8%)	3958 (55.3%)	10,799 (54.9%)	<0.0001	1626 (35.7%)	1310 (39.5%)	1461 (38.6%)	4397 (37.7%)	0.0010	<0.0001
1668 (26.6%)	1167 (18.7%)	1649 (23.1%)	4484 (22.8%)	<0.0001	884 (19.4%)	504 (15.2%)	710 (18.8%)	2098 (18.0%)	<0.0001	<0.0001
4726 (75.3%)	5142 (82.5%)	5822 (81.4%)	15,690 (79.8%)	<0.0001	3027 (66.5%)	2282 (68.9%)	2672 (70.7%)	7981 (68.5%)	0.0002	<0.0001
3676 (58.5%)	3992 (64.1%)	4307 (60.2%)	11,975 (60.9%)	<0.0001	2205 (48.4%)	1782 (53.8%)	1955 (51.7%)	5942 (51.0%)	<0.0001	<0.0001
1867 (29.7%)	1398 (22.4%)	1540 (21.5%)	4805 (24.4%)	<0.0001	1862 (40.9%)	1170 (35.3%)	1211 (32.0%)	4243 (36.4%)	<0.0001	<0.0001
2017 (32.1%)	1775 (28.5%)	2316 (32.4%)	6108 (31.1%)	<0.0001	1361 (29.9%)	877 (26.5%)	1139 (30.1%)	3377 (29.0%)	0.0008	0.0001
1485 (23.6%)	1298 (20.8%)	1681 (23.5%)	4464 (22.7%)	0.0001	1333 (29.3%)	934 (28.2%)	1138 (30.1%)	3405 (29.2%)	0.21	<0.0001
1 (0.0%)	3 (0.0%)	1 (0.0%)	5 (0.0%)	NA	3077 (67.6%)	2187 (66.0%)	2522 (66.7%)	7786 (66.8%)	0.33	NA
NA	NA	NA	NA	NA	1295 (28.4%)	832 (25.1%)	921 (24.4%)	3048 (26.2%)	<0.0001	NA
3593 (57.2%)	4306 (69.1%)	4717 (66.0%)	12,616 (64.2%)	<0.0001	2284 (50.2%)	1947 (58.8%)	2064 (54.6%)	6295 (54.0%)	<0.0001	<0.0001
				<0.0001					0.14	<0.0001
1397 (22.2%)	1403 (22.5%)	1648 (23.0%)	4448 (22.6%)		1073 (23.6%)	775 (23.4%)	943 (24.9%)	2791 (24.0%)		
3032 (48.3%)	3213 (51.6%)	3717 (52.0%)	9962 (50.7%)		1916 (42.1%)	1425 (43.0%)	1611 (42.6%)	4952 (42.5%)		
1557 (24.8%)	1373 (22.0%)	1550 (21.7%)	4480 (22.8%)		1208 (26.5%)	878 (26.5%)	991 (26.2%)	3077 (26.4%)		
294 (4.7%)	243 (3.9%)	237 (3.3%)	774 (3.9%)		356 (7.8%)	235 (7.1%)	237 (6.3%)	828 (7.1%)		

### Renal outcomes and exercise

**Fig. 2:**
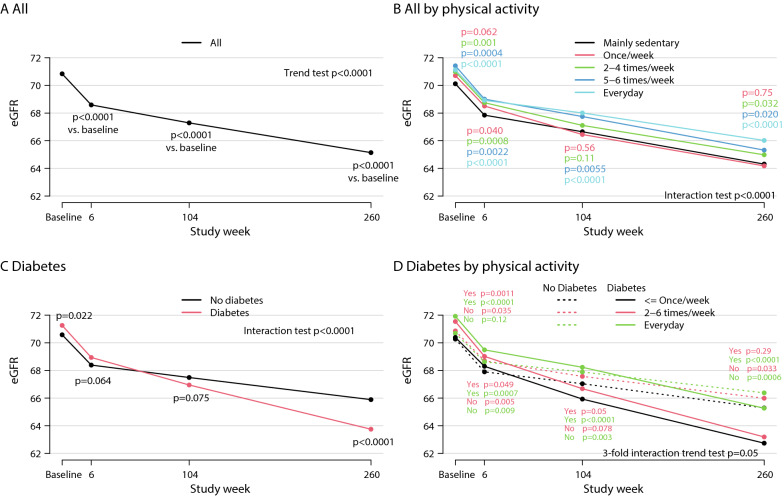
Decline of estimated glomerular filtration rate (eGFR) in all patients (**A**), all patients categorized by physical activity (**B**), patients with diabetes or no diabetes (**C**) and diabetes or no diabetes categorized by physical activity (**D**)

Figure 2 displays the time course of eGFR slopes in the overall population (Fig. 2A, all), categorized by five groups of physical activity (Fig. 2B), categorized by diabetes status (Fig. 2C) and categorized by diabetes status and physical activity in these exercise groups (Fig. 2D). There was a significant decline of eGFR in the overall population at 6, 104 and 260 weeks compared to baseline (p for trend < 0.0001) (Fig. 2A). When categorized by physical activity, there was no difference in decline of eGFR between “once/week” and “mainly sedentary” (yearly decline averaged over 5 years 1.31 (1.19–1.42) vs. 1.16(1.08–1.25) ml/min/1.73 m^2^ (p = 0.56 at week 104; p = 0.75 at week 260). In marked contrast, significantly smaller declines were observed in patients with 5–6 times/week and every day physical activity compared with mainly sedentary individuals (Fig. 2B) with a significant interaction between physical activity and time (interaction test p < 0.0001) indicating that the differences between activity categories increase over time. Figure 2C shows a significantly larger decline of eGFR over time in patients with diabetes compared to those without diabetes (interaction test p < 0.0001). There was a significant difference of eGFR at week 260 (p < 0.0001, Fig. 2C). Figure 2D shows the interaction between physical activity levels in patients with and without diabetes and the eGFR profiles. In the overall population, in patients without diabetes there was a smaller yearly decline in eGFR at every day activity compared to less active patients (p = 0.037). The yearly decline in kidney function was more pronounced in patients with diabetes than without (p < 0.0001 for all exercise levels). Patients with diabetes who were active every day had less yearly eGFR decline than those with ≤ once a week activity (p = 0.0004) and achieved a similar level of eGFR compared to sedentary patients without diabetes. The detailed eGFR data with ranges are summarized in Table 2 and the detailed eGFR changes are shown in Additional file 1: Fig. S2.

**Table 2:** Renal function over time by physical activity groups and diabetes status

	Baseline		Week 6		Week 104		Week 260	
	Mean eGFR (min.–max.)	p-value	Mean eGFR (min.–max.)	p-value	Mean eGFR (min.–max.)	p-value	Mean eGFR (min.–max.)	p-value
All	70.84 (70.66–71.01)	Ref.	68.59 (68.42–68.77)	<0.0001	67.29 (67.11–67.48)	<0.0001	65.14 (64.93–65.34)	<0.0001
All by physical activity								
Mainly sedentary	70.12 (69.76–70.49)	Ref.	67.85 (67.48–68.22)	Ref.	66.65 (66.25–67.04)	Ref.	64.31 (63.86–64.75)	Ref.
Once/week	70.71 (70.21–71.22)	0.062	68.51 (68.00–69.03)	0.04	66.45 (65.90–67.00)	0.56	64.18 (63.57–64.79)	0.75
2–4 times/week	70.99 (70.63–71.35)	0.001	68.75 (68.38–69.11)	0.0008	67.11 (66.72–67.49)	0.11	64.98 (64.56–65.40)	0.032
5–6 times/week	71.42 (70.80–72.04)	0.0004	69.01 (68.37–69.64)	0.0022	67.75 (67.09–68.41)	0.0055	65.32 (64.60–66.05)	0.02
Everyday	71.12 (70.82–71.41)	<0.0001	68.92 (68.63–69.22)	<0.0001	68.01 (67.70–68.32)	<0.0001	66.02 (65.69–66.36)	<0.0001
By diabetes								
No diabetes	70.58 (70.31–70.86)	Ref.	68.39 (68.11–68.67)	Ref.	67.49 (67.20–67.77)	Ref.	65.89 (65.58–66.19)	Ref.
Diabetes	71.26 (70.86–71.66)	0.022	68.94 (68.53–69.35)	0.064	66.95 (66.53–67.36)	0.075	63.75 (63.31–64.19)	<0.0001
Diabetes by physical activity								
No diabetes, ≤ once/week	70.27 (69.85–70.69)	Ref.	67.90 (67.47–68.33)	Ref.	67.04 (66.59–67.49)	Ref.	65.29 (64.80–65.78)	Ref.
No diabetes, 2–6 times/week	70.86 (70.44–71.28)	0.035	68.70 (68.27–69.13)	0.005	67.57 (67.12–68.01)	0.078	65.99 (65.51–66.47)	0.033
No diabetes, everyday	70.69 (70.30–71.09)	0.12	68.62 (68.22–69.03)	0.009	67.89 (67.47–68.31)	0.003	66.38 (65.93–66.83)	0.0006
Diabetes, ≤ once/week	70.39 (69.84–70.93)	Ref.	68.30 (67.75–68.86)	Ref.	65.93 (65.35–66.51)	Ref.	62.74 (62.10–63.37)	Ref.
Diabetes, 2–6 times/week	71.54 (70.95–72.14)	0.0011	69.02 (68.41–69.62)	0.049	66.68 (66.05–67.31)	0.05	63.19 (62.50–63.87)	0.029
Diabetes, everyday	71.92 (71.35–72.48)	<0.0001	69.49 (68.92–70.07)	0.0007	68.23 (67.63–68.82)	<0.0001	65.27 (64.63–65.92)	<0.0001

**Fig. 3:**
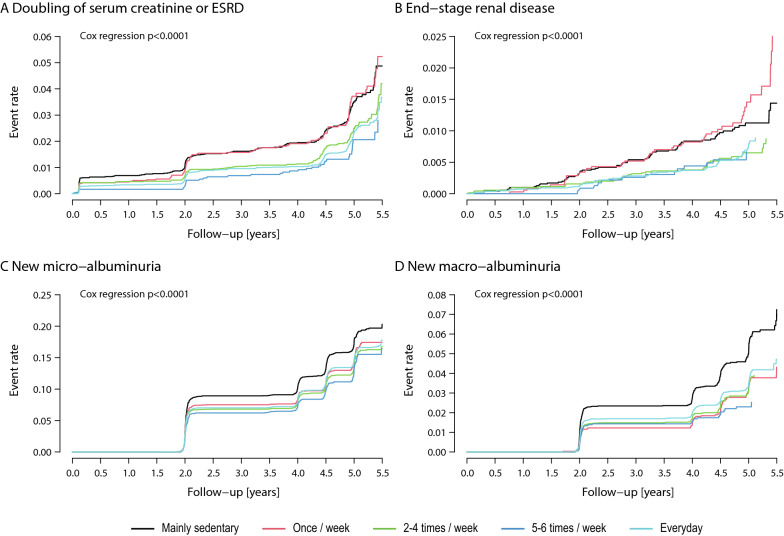
Cumulative incidence for doubling of serum creatinine or end-stage renal disease (ESRD) (**A**), end-stage renal disease (ESRD) (**B**), new microalbuminuria (**C**) and new macroalbuminuria (**D**) according to physical activity level

Figure 3 displays the incidence of the composite renal outcome “doubling of serum creatinine or end-stage renal disease” (ESRD) (Fig. 3A), of ESRD (Fig. 3B), of new micro-albuminuria (Fig. 3C) and new macro-albuminuria (Fig. 3D). There was an overall association of physical activity levels with the renal outcomes (Figs. 3A, B) (p < 0.0001) and with new micro- or macro-albuminuria (Fig. 3C, D) (p < 0.0001). For the renal outcomes (Fig. 3A, B), physical activity levels of “2–4 times/week” or “every day” were associated with lower risk, while there was no striking difference between “mainly sedentary” patients and patients with reported “once a week” physical activity. For albuminuria outcomes all activity groups with physical activity showed fewer events than the “mainly sedentary” category (Fig. 3C, D).

### Renal outcomes and diabetes

**Fig. 4:**
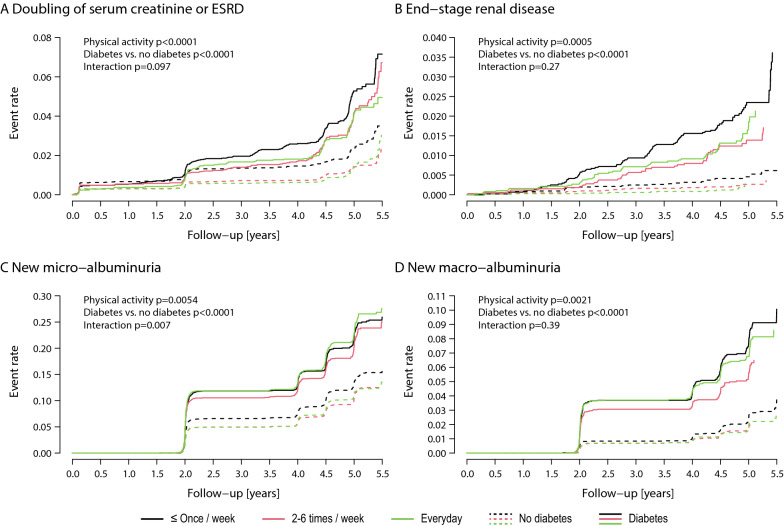
Cumulative incidence for doubling of serum creatinine or end-stage renal disease (ESRD) (**A**), end-stage renal disease (ESRD) (**B**), new microalbuminuria (**C**) and new macroalbuminuria (**D**) in patients with or without diabetes according to physical activity level

Figure 4 shows the association of physical exercise combined into three categories (≤ once/week, 2–6 times/week, everyday) with renal and albuminuria endpoints in patients with and without diabetes. There was a significant association of physical activity and of diabetes with the composite of doubling of serum creatinine or ESRD (both p < 0.0001), which was consistent in those with or without diabetes (p = 0.097) (Fig. 4A). Similar results were obtained for ESRD (Fig. 4B) with a significant and independent effect of physical activity (p = 0.0005) and diabetes (p < 0.0001). Figure 4C summarizes the incidences of new microalbuminuria and Fig. 4D of new macro-albuminuria. New microalbuminuria was inversely associated with physical activity (p = 0.0054) and with diabetes status (p < 0.0001). However, there was a significant interaction between the two (p = 0.0068). New macro-albuminuria was also associated inversely with physical activity (p = 0.0021) and diabetes status (p < 0.0001).

Additional file 1: Fig. S1 (left) shows the hazard ratios of activity levels (using “≥ once/week” as reference) separated for patients with or without diabetes regarding the composite of doubling of serum creatinine or ESRD in an unadjusted analysis. The HRs indicate that physically active patients benefit irrespective of diabetes status. However, when adjusting for relevant clinical conditions, the benefit was less and no longer significant (Additional file 1: Fig. S1, right).

### Cardiovascular outcomes

The association of physical activity with CV outcomes was investigated to demonstrate consistency with the literature. For the fourfold primary endpoint of the original studies (CV death, myocardial infarction, stroke or hospitalization for heart failure) there was an overall effect of physical activity levels (p < 0.0001) with a striking reduction of CV risk with any level of physical activity more than once/week (Additional file 1: Fig. S2A); between sedentary or once/week activity there was no difference. Similar results were obtained for CV death (p < 0.0001) (Additional file 1: Fig. S2B), not for myocardial infarction (p = 0.14) (Additional file 1: Fig. S2C), but for stroke and hospitalization for heart failure (p < 0.0001 for both) (Additional file 1: Fig. S2D, E). When patients were categorized according to physical activity (three levels) and diabetes versus no diabetes, there was a significant effect for the fourfold endpoint (p < 0.0001) (Additional file 1: Fig. S3A) and CV death (p < 0.0001) of exercise and diabetes status (Additional file 1: Fig. S3B). There was no interaction between physical activity and diabetes status (p = 0.68 for the fourfold primary endpoint and p = 0.32 for CV death) indicating an independence of physical activity and diabetes status. For myocardial infarction there were only minor differences between activity levels (p = 0.090) (Additional file 1: Fig. S3C) but diabetes had a clearly detrimental effect (p < 0.0001). Less physical activity and diabetes status were predictive for stroke and hospitalization for congestive heart failure (CHF) (p < 0.0001 for both, Additional file 1: Fig. S3D, E). As no interaction between physical activity and diabetes status was detected (p = 0.89 for stroke, p = 0.11 for CHF hospitalization), both effects are additive. Thus, for the majority of CV outcomes, diabetes and physical activity levels were independently associated with risk. Additional file 1: Fig. S4 shows the hazard for the fourfold primary endpoint (Additional file 1: Fig. S3A) and CV death (Additional file 1: Fig. S3B) unadjusted (left) and adjusted (right). Greater physical activity was associated with a reduced risk for the fourfold primary endpoint and to CV death in the unadjusted and adjusted analyses.

## Discussion

ONTARGET/TRANSCEND investigated the effect of telmisartan, ramipril or both on CV and renal outcomes. As self-reported activity levels were rigorously captured at baseline, and history of diabetes was an inclusion criterion, this database offered the unique opportunity to examine the association of self-reported exercise intensity with CV and renal outcomes in patients with or without diabetes within the context of a clinical trial with rigorously adjudicated endpoints. Indeed, the present study suggests that more intensive physical activity was associated with less renal complications. The typical primary renal outcome of kidney outcome trials, (the composite of doubling of serum-creatinine and ESRD), as well as ESRD itself, were less frequently observed with higher versus lower levels of physical exercise. The same association was also found for new onset of micro- or macro-albuminuria. These associations were present irrespective of diabetes but as expected, renal outcomes were far more frequent in those with diabetes. Daily exercise relative to lower levels of exercise was also associated with a reduction in rate of the yearly decline in eGFR.

In the present analysis, a relative risk reduction of 43% was found for the composite renal outcome at activity levels of 2–6 times/week and of 44% at every day versus inactive people. These data suggest that at least moderate activity is necessary to provide benefit on renal outcomes. Previous small studies, have shown that physical inactivity is associated with worse kidney outcomes [18]. A meta-analysis compiling data from small observational studies, reported comparable findings to our study [3]. These studies indicated a relative risk reduction of 18% for the renal composite outcome comparing high versus low physical activity, however, the quality of contributory evidence was low for these small observational studies [3].

Physical activity is also beneficial in the general population where it is associated with weight loss and lower blood pressure [19]. The KDIGO Clinical Practice Guidelines recommend physical activity for a cumulative duration of at least 150 min per week to reduce blood pressure and CV events [10]. This exercise level is not achieved by two thirds of the adults in the USA [20]. These findings extend those data to high-risk patients who already had an event or had diabetes with proven vascular disease. Nevertheless, adjustment of clinical covariants neutralized some of the effects indicating that the benefit of exercise at large is not independent from the CV risk predictors. Furthermore, there may be unknown confounders. People at high CV risk enrolled in an outcome trial and engaging in intensive exercise may also adapt other behaviors towards a healthier lifestyle.

CV outcomes were also associated with physical activity level. Thus, the present analysis supports prior studies reporting less CV outcomes such as heart failure hospitalization [21] and coronary events [22] with greater intensity of exercise. A small lifestyle and exercise intervention study indicated an improvement of diastolic myocardial function and a reduced rate of CKD progression with that lifestyle intervention [23]. The effects of exercise on renal function and CV outcomes remained significant after adjustment for covariants indicative of independent effects of exercise on renal function. In a large cohort of patients, there was an association of self-reported exercise (low, moderate, high) in individuals starting at age < 20 years with a small risk reduction of CKD. This is in line with our study, but a separation between diabetes and no diabetes was not done in this non-diseased population [24]. In elderly patients, the Atherosclerosis Risk in Communities (ARIC) study showed also a reduction of developing CKD in active participants [25]. Our study extends those findings by looking at the slope of eGFR, which might be more sensitive as a clinical renal endpoint must not be achieved and evaluated patients at particular high risk after a stroke, myocardial infarction or with proven atherosclerotic disease in individuals with and without diabetes.

One might speculate that physical activity would be especially effective in patients with diabetes since physical activity improves insulin sensitivity, endothelial function [26–29], cellular senescence [30] and interstitial fibrosis [31], which all are suggested to facilitate end-organ damage and renal dysfunction in diabetes [4–6]. Our data suggest that in those with diabetes, there was a modest association of physical activity with better renal outcomes. Physical activity was also associated with a smaller decline of eGFR and less new onset albuminuria. Over almost 5 years, the eGFR loss, in those with diabetes who undertook intensive exercise, equalled the eGFR loss in sedentary non-diabetic patients. Since patients in ONTARGET/TRANSCEND were on a RAAS-inhibitor therapy consisting of telmisartan, ramipril or both, these effects on exercise appear to be additive to a background of RAAS inhibition [15, 16].

Our study had some limitations. This was a post-hoc observational analysis, studying the association of self-reported exercise in the absence or presence of diabetes, and as such, exercise level was not subject to randomization and this analysis could suffer from unmeasured confounding. Furthermore, self-reported exercise could create some sources of unreliability. However, the large number of patients and the rigorously captured renal parameters in the context of a large scale clinical trial, and the evaluation of five groups of self-reported exercise levels in an adequately powered study has enabled an analysis of the association of physical activity and renal and CV endpoints with great rigour than previously possible. Physical activity levels were only captured at baseline and changes over time could have modified outcomes. Data did not account for specific diets, which could have affected renal function. This study does not inform about the association of physical activity and kidney function in more advanced CKD as these patients were excluded from ONTARGET/TRANSEND [15, 16].

## Conclusions

These data support current recommendations [11, 32] encouraging regular physical activity because exercise intensity was associated with beneficial CV and renal outcomes, from a threshold physical activity level of more than two exercise sessions per week. The benefits of activity levels on renal and CV outcomes were seen in patients with and without diabetes. This observation provides a strong evidential basis for prospectively conducting an adequately powered RCT to formally evaluate the effects of physical activity or even exercise training programs on renal and CV outcomes, which co-occur so frequently.

## Supplementary Information

**Additional file1**: **Figure S1** Hazard ratios for doubling of serum creatinine and end-stage renal disease (ESRD) according to physical activity in patients with and without diabetes in unadjusted (right) and adjusted (left) analysis. The analyses on the right were adjusted for the variables diastolic blood pressure (DBP), baseline systolic blood pressure (SBP), heart rate (HR), age, sex, body mass index, renal function, geographical region, physical activity, formal education, alcohol consumption, tobacco use, history of hypertension, myocardial infarction, stroke, transient ischemic attack, heart rhythm, comedications, study and study medications. **Figure S2** Cumulative incidence for the fourfold primary endpoint (cardiovascular death, myocardial infarction, stroke, hospitalization for heart failure worsening) (A), cardiovascular death (B), myocardial infarction (C), stroke (D) and hospitalization for heart failure worsening (E) according to physical activity level. **Figure S3** Cumulative incidence for fourfold primary endpoint (cardiovascular death, myocardial infarction, stroke, hospitalization for heart failure worsening) (A), cardiovascular death (B), myocardial infarction (C), stroke (D) and hospitalization for heart failure worsening (E) according to physical activity level in patients with or without diabetes. **Figure S4** Hazard ratios for the fourfold primary endpoint (A, cardiovascular death, myocardial infarction, hospitalization for heart failure worsening) and cardiovascular death (B) in patients with or without diabetes according to physical activity in unadjusted (left) and adjusted (right) analysis. The analyses on the right were adjusted for the variables diastolic blood pressure (DBP), baseline systolic blood pressure (SBP), heart rate (HR), age, sex, body mass index, renal function, geographical region, physical activity, formal education, alcohol consumption, tobacco use, history of hypertension, myocardial infarction, stroke, transient ischemic attack, heart rhythm, comedications, study and study medications.

## Data Availability

All data generated in this analysis are included in this article and in the Additional files.
