# Supplementary material for: Association between exercise frequency with renal and cardiovascular outcomes in diabetic and non-diabetic individuals at high cardiovascular risk
Source: Cardiovasc Diabetol. 2022 Jan 20;21:12. doi: 10.1186/s12933-021-01429-w (PMC8772075; doi:10.1186/s12933-021-01429-w)
Supplement: Supplementary file 1 — Additional file1: Figure S1 Hazard ratios for doubling of serum creatinine and end-stage renal disease (ESRD) according to physical activity in patients with and without diabetes in unadjusted (right) and adjusted (left) analysis. The analyses on the right were adjusted for the variables diastolic blood pressure (DBP), baseline systolic blood pressure (SBP), heart rate (HR), age, sex, body mass index, renal function, geographical region, physical activity, formal education, alcohol consumption, tobacco use, history of hypertension, myocardial infarction, stroke, transient ischemic attack, heart rhythm, comedications, study and study medications. Figure S2 Cumulative incidence for the fourfold primary endpoint (cardiovascular death, myocardial infarction, stroke, hospitalization for heart failure worsening) (A), cardiovascular death (B), myocardial infarction (C), stroke (D) and hospitalization for heart failure worsening (E) according to physical activity level. Figure S3 Cumulative incidence for fourfold primary endpoint (cardiovascular death, myocardial infarction, stroke, hospitalization for heart failure worsening) (A), cardiovascular death (B), myocardial infarction (C), stroke (D) and hospitalization for heart failure worsening (E) according to physical activity level in patients with or without diabetes. Figure S4 Hazard ratios for the fourfold primary endpoint (A, cardiovascular death, myocardial infarction, hospitalization for heart failure worsening) and cardiovascular death (B) in patients with or without diabetes according to physical activity in unadjusted (left) and adjusted (right) analysis. The analyses on the right were adjusted for the variables diastolic blood pressure (DBP), baseline systolic blood pressure (SBP), heart rate (HR), age, sex, body mass index, renal function, geographical region, physical activity, formal education, alcohol consumption, tobacco use, history of hypertension, myocardial infarction, stroke, transient ischemic attack, h [file 12933_2021_1429_MOESM1_ESM.pdf]

Supplement figure 1

Hazard ratios for physical activity according to diabetes status (no ● / yes ●)

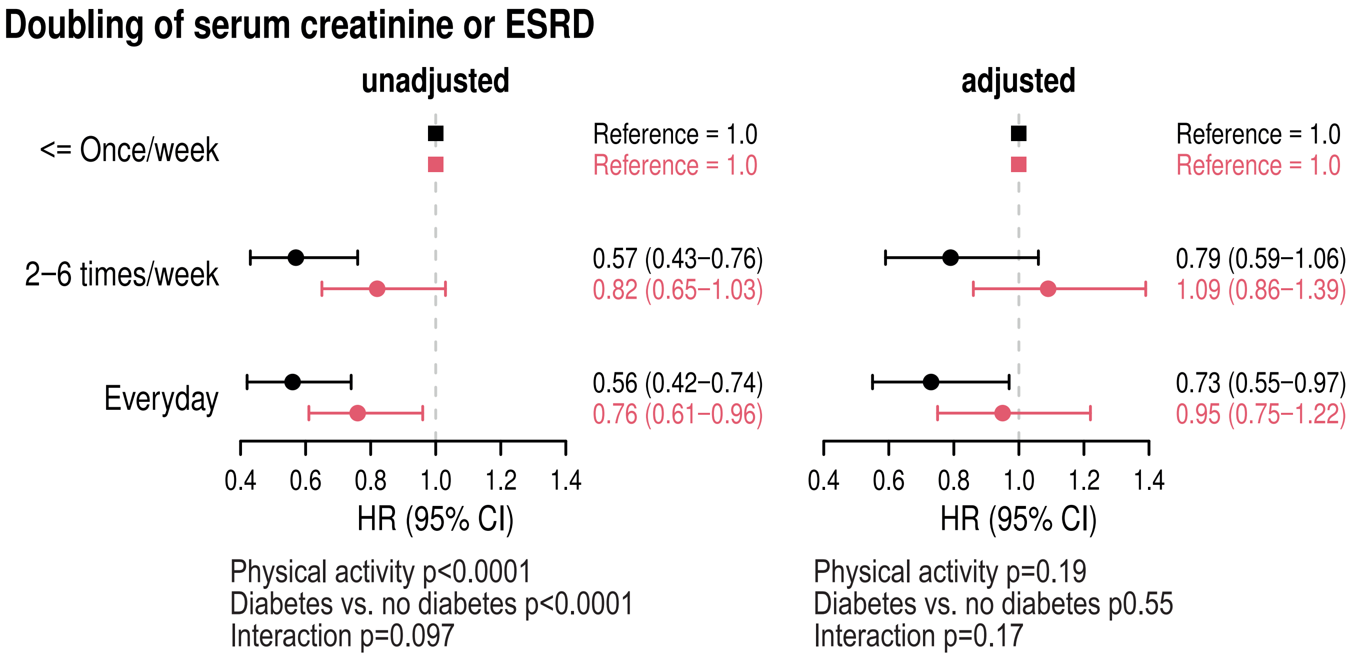

Supplement figure 2

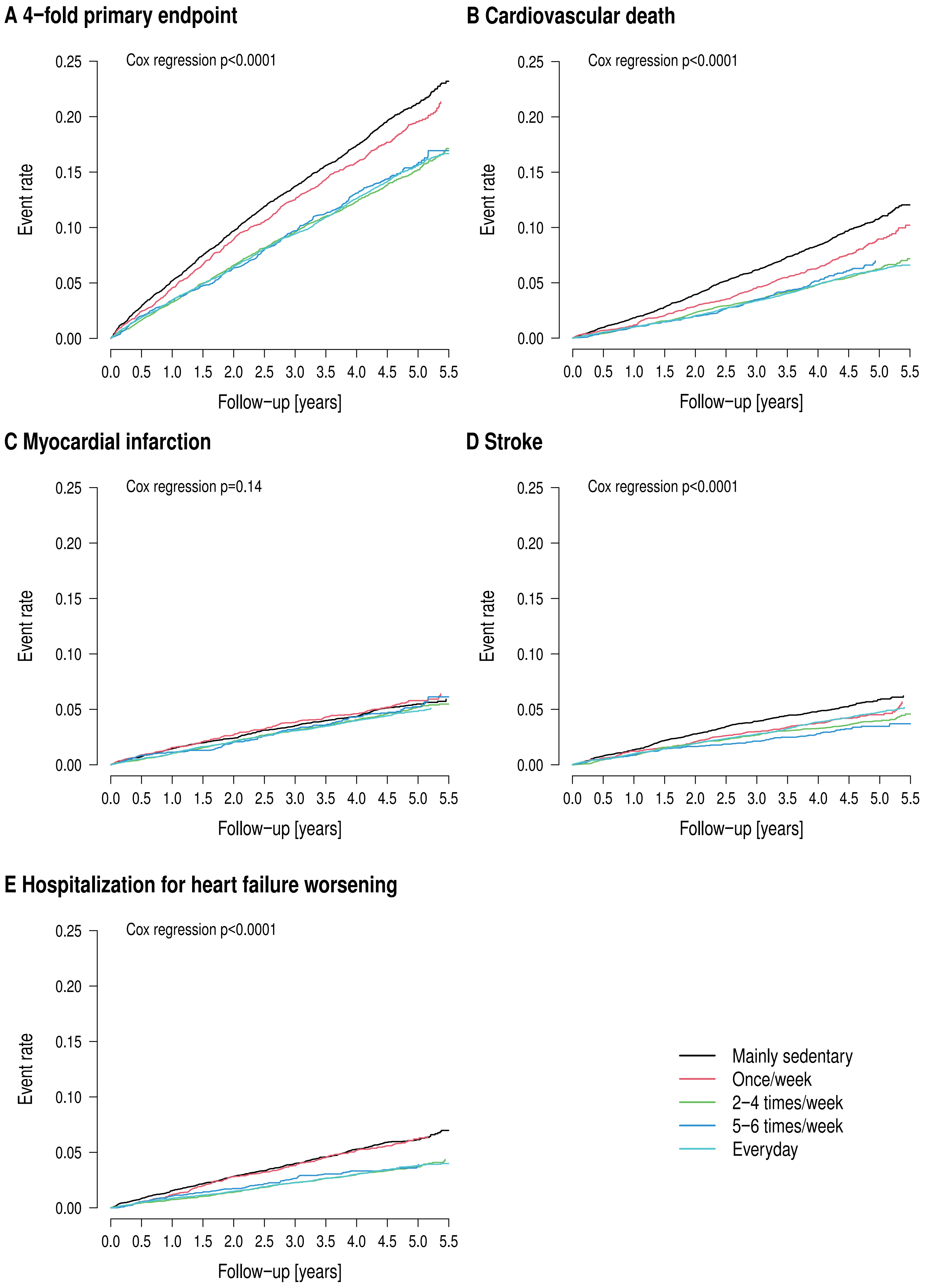

Supplement figure 3

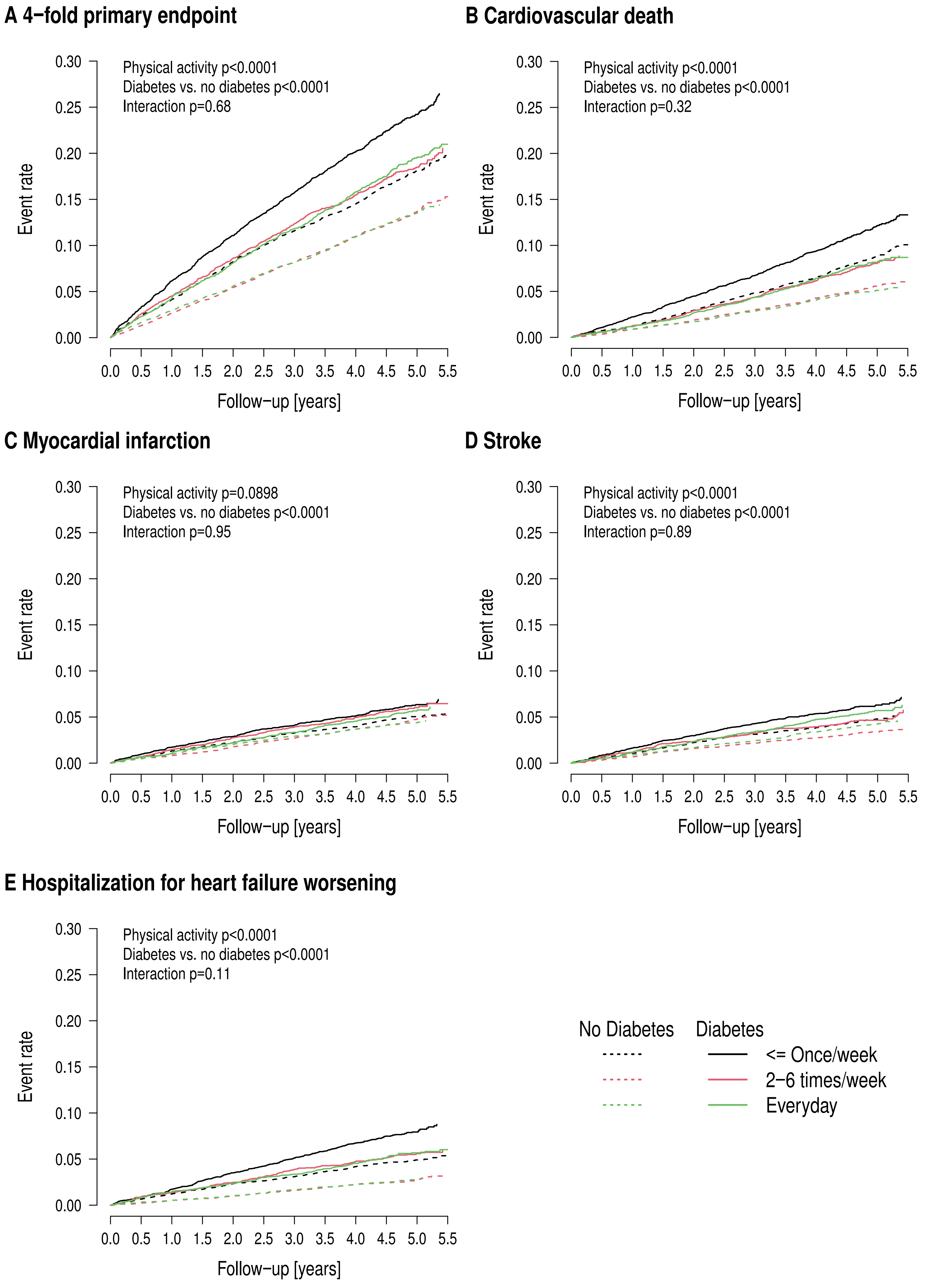

Supplement figure 4

Hazard ratios for physical activity according to diabetes status (no ● / yes ●)

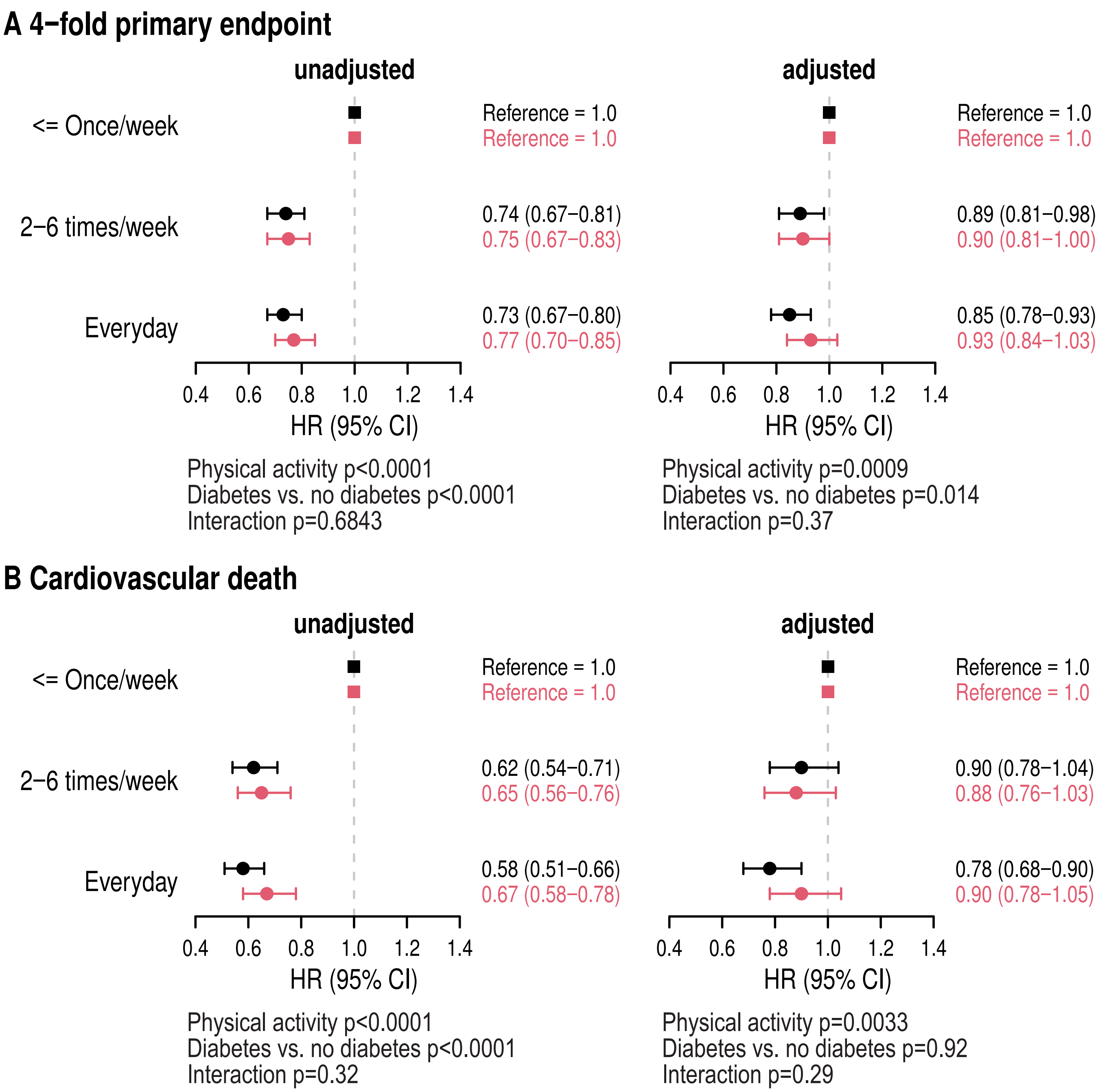

Supplement table 1: Differences in estimated glomerular filtration rate (eGFR) according to exercise levels and diabetic status

| | Baseline | | Week 6 | | Week 104 | | Week 260 | |
|---|---|---|---|---|---|---|---|---|
| | Mean (95%CI) | p-value | Mean (95%CI) | p-value | Mean (95%CI) | p-value | Mean (95%CI) | p-value |
| Diff vs. Baseline | | | -2.24 (-2.37, -2.11) | <0.0001 | -3.54 (-3.70, -3.38) | <0.0001 | -5.70 (-5.89, -5.51) | <0.0001 |
| Diff vs. Mainly sedentary | | | | | | | | |
| Once/week | 0.59 (-0.03, 1.21) | 0.0624 | 0.66 (0.03, 1.30) | 0.0404 | -0.20 (-0.87, 0.48) | 0.5648 | -0.12 (-0.87, 0.63) | 0.7493 |
| 2-4 times/week | 0.87 (0.35, 1.38) | 0.0010 | 0.90 (0.37, 1.42) | 0.0008 | 0.46 (-0.10, 1.01) | 0.1059 | 0.67 (0.06, 1.29) | 0.0322 |
| 5-6 times/week | 1.30 (0.58, 2.02) | 0.0004 | 1.16 (0.42, 1.90) | 0.0022 | 1.10 (0.32, 1.88) | 0.0055 | 1.02 (0.16, 1.87) | 0.0197 |
| Everyday | 0.99 (0.52, 1.47) | <0.0001 | 1.08 (0.59, 1.56) | <0.0001 | 1.36 (0.85, 1.87) | <0.0001 | 1.72 (1.16, 2.28) | <0.0001 |
| Diff vs no diabetes | 0.68 (0.10, 1.26) | 0.0218 | 0.55 (-0.03, 1.13) | 0.0642 | -0.54 (-1.14, 0.06) | 0.0754 | -2.14 (-2.76, -1.51) | <0.0001 |
| No Diabetes; Diff vs ≤ Once/week | | | | | | | | |
| 2-6 times/week | 0.59 (0.04, 1.14) | 0.0353 | 0.80 (0.24, 1.36) | 0.0051 | 0.53 (-0.06, 1.12) | 0.0779 | 0.70 (0.06, 1.35) | 0.0329 |
| Everyday | 0.42 (-0.11, 0.95) | 0.1188 | 0.72 (0.18, 1.27) | 0.0089 | 0.85 (0.28, 1.42) | 0.0034 | 1.10 (0.47, 1.72) | 0.0006 |
| Diabetes; Diff vs ≤ Once/week | | | | | | | | |
| 2-6 times/week | 1.15 (0.46, 1.85) | 0.0011 | 0.71 (0.00, 1.42) | 0.0486 | 0.74 (-0.01, 1.49) | 0.0531 | 0.45 (-0.39, 1.29) | 0.2926 |
| Everyday | 1.53 (0.86, 2.20) | <0.0001 | 1.19 (0.51, 1.88) | 0.0007 | 2.29 (1.57, 3.02) | <0.0001 | 2.54 (1.73, 3.35) | <0.0001 |
